# Supplementary material for: Phase angle is related to oxidative stress and antioxidant biomarkers in breast cancer patients undergoing chemotherapy
Source: PLoS One. 2023 Jun 8;18(6):e0283235. doi: 10.1371/journal.pone.0283235 (PMC10249836; doi:10.1371/journal.pone.0283235)
Supplement: Attachment — Submitted filename: Response to Reviewers (Final).docx [file pone.0283235.s001.docx]

Thank you very much for the opportunity to review our manuscript “**Phase angle is related to oxidative stress and antioxidant biomarkers in breast cancer patients undergoing chemotherapy”**. We believe that all reviewer suggestions have substantially contributed to a better presentation of our findings. We thank them for their time and expertise.

Please find below a point-by-point response to reviewer’s comments.

**JOURNAL REQUIREMENTS:**

When submitting your revision, we need you to address these additional requirements.

1. Please ensure that your manuscript meets PLOS ONE's style requirements, including those for file naming. The PLOS ONE style templates can be found at

<https://journals.plos.org/plosone/s/file?id=wjVg/PLOSOne_formatting_sample_main_body.pdf>

and

<https://journals.plos.org/plosone/s/file?id=ba62/PLOSOne_formatting_sample_title_authors_affiliations.pdf>

This manuscript meets PLOS ONE's style requirements, including those for file naming.

2. Please include your full ethics statement in the ‘Methods’ section of your manuscript file. In your statement, please include the full name of the IRB or ethics committee who approved or waived your study, as well as whether or not you obtained informed written or verbal consent. If consent was waived for your study, please include this information in your statement as well.

The Institutional Review Board at the University of Sao Paulo, General Hospital, approved the current study, obtained informed written consent, according to guidelines and standards for research involving human beings, regulated by Resolution 466/12 of the National Health Council (Protocol: HCRP 14608/2017).

3. Thank you for stating the following financial disclosure:

“Bruna Ramos da Silva was founded by São Paulo Research Foundation (FAPESP). Grant number: 2017/07963-0 and FAPESP fellowship Grant number: 2019/09877-9”

Please state what role the funders took in the study. If the funders had no role, please state: "The funders had no role in study design, data collection and analysis, decision to publish, or preparation of the manuscript."

If this statement is not correct you must amend it as needed.

BRS was founded by São Paulo Research Foundation (FAPESP). Grant number: 2017/07963-0 and FAPESP fellowship Grant number: 2019/09877-9. The funders had no role in study design, data collection and analysis, decision to publish, or preparation of the manuscript.

4. In your Data Availability statement, you have not specified where the minimal data set underlying the results described in your manuscript can be found. PLOS defines a study's minimal data set as the underlying data used to reach the conclusions drawn in the manuscript and any additional data required to replicate the reported study findings in their entirety. All PLOS journals require that the minimal data set be made fully available. For more information about our data policy, please see http://journals.plos.org/plosone/s/data-availability.

Upon re-submitting your revised manuscript, please upload your study’s minimal underlying data set as either Supporting Information files or to a stable, public repository and include the relevant URLs, DOIs, or accession numbers within your revised cover letter. For a list of acceptable repositories, please see http://journals.plos.org/plosone/s/data-availability#loc-recommended-repositories. Any potentially identifying patient information must be fully anonymized.

Important: If there are ethical or legal restrictions to sharing your data publicly, please explain these restrictions in detail. Please see our guidelines for more information on what we consider unacceptable restrictions to publicly sharing data: http://journals.plos.org/plosone/s/data-availability#loc-unacceptable-data-access-restrictions. Note that it is not acceptable for the authors to be the sole named individuals responsible for ensuring data access.

We will update your Data Availability statement to reflect the information you provide in your cover letter.

For resubmission of our revised manuscript we include uploading the minimal set of underlying data set as supporting information files.

**REVIEWERS' COMMENTS:**

Reviewer's Responses to Questions

Comments to the Author

1. Is the manuscript technically sound, and do the data support the conclusions?

The manuscript must describe a technically sound piece of scientific research with data that supports the conclusions. Experiments must have been conducted rigorously, with appropriate controls, replication, and sample sizes. The conclusions must be drawn appropriately based on the data presented.

Reviewer #1: Yes

Reviewer #2: Partly

Thanks for the appreciation.

2. Has the statistical analysis been performed appropriately and rigorously?

Reviewer #1: No

Reviewer #2: No

Thanks to both reviewers. In the next topics we hope to explain in more detail.

3. Have the authors made all data underlying the findings in their manuscript fully available?

The PLOS Data policy requires authors to make all data underlying the findings described in their manuscript fully available without restriction, with rare exception (please refer to the Data Availability Statement in the manuscript PDF file). The data should be provided as part of the manuscript or its supporting information, or deposited to a public repository. For example, in addition to summary statistics, the data points behind means, medians and variance measures should be available. If there are restrictions on publicly sharing data—e.g. participant privacy or use of data from a third party—those must be specified.

Reviewer #1: Yes

Reviewer #2: Yes

Thanks for the appreciation.

4. Is the manuscript presented in an intelligible fashion and written in standard English?

PLOS ONE does not copyedit accepted manuscripts, so the language in submitted articles must be clear, correct, and unambiguous. Any typographical or grammatical errors should be corrected at revision, so please note any specific errors here.

Reviewer #1: Yes

Reviewer #2: Yes

The manuscript has been proofread for the English text.

5. Review Comments to the Author

Please use the space provided to explain your answers to the questions above. You may also include additional comments for the author, including concerns about dual publication, research ethics, or publication ethics. (Please upload your review as an attachment if it exceeds 20,000 characters)

**REVIEWER 1**

Reviewer #1: The authors aimed to examine the association between PhA and inflammatory and oxidative stress biomarkers in women with breast cancer before and after one month of chemotherapy. The authors should be congratulated by the relevance of the research. However, several methodological issues should be addressed:

1. Please clarify if inclusion criteria involves active smoking status, metabolic syndrome, hypertension.

The inclusion criteria for this study were detailed:

- Inclusion criteria: women treated in the hospital during the follow-up, age ≥18 years and <65 years; a histologically confirmed diagnosis of early breast cancer (range of stage I – III); and very first chemotherapy treatment course.

- Exclusion criteria: smokers, patients with MetS, with worse blood pressure control, which means the use of two or more antihypertensive drugs, lipid disorders, which means values above the normal range for triglycerides, total cholesterol and low dense cholesterol according to the criteria of the National Cholesterol Education Program’sAdult Treatment Panel III (NCEP-ATP III) [44] and, the Brazilian nation recommendations [45], with any type of diabetes (type I, type II, gestational) or with a more recent glucose test above 125 mg/dl according to the results available on the electronic clinical records; who previously has already received or started chemotherapy in any other moment of life; those fitted with a defibrillator, cardiac pacemaker, metal implants or those with a local infection/wound preventing the use of bioelectric impedance analysis pads, those unable to use a handheld dynamometer due to a neuromuscular disorder.

2. More details about the recruitment and screening process of the control group are required (ex, where were they recruited from? Other medical appoitments? How was the physical level of CG females? Did this women also present the metabolic syndrome and its features and were active smokers?

Women in control groups were recruited at the same hospital, the participants were employees or graduate student from the School of Medicine of Ribeirao Preto, Sao Paulo, Brazil. For this group potential participants were weighed and measured to determine BMI and completed a Health Status Screening Form to determine if they had any prior cancer or were under hormone or any other medication which could modify the metabolism that would have excluded them from participating in the study. The inclusion for this group were: female (sex-matched), > 18 years old <60 years old, no history of cancer and exclusion criteria were the same applied to the breast cancer group: smokers, patients with MetS, with worse blood pressure control, which means the use of two or more antihypertensive drugs, lipid disorders, which means values above the normal range for triglycerides, total cholesterol and low dense cholesterol according to the criteria of the National Cholesterol Education Program’sAdult Treatment Panel III (NCEP-ATP III) [44] and, the Brazilian nation recommendations [45], with any type of diabetes (type I, type II, gestational) or with a more recent glucose test above 125 mg/dl according to the results available on the electronic clinical records, HIV, thyroid disease that is not currently managed with medication, pregnancy, BIA exclusion facto. After screening, the participants eligible for the study were scheduled for the data collection visit.

3. Before describing the methods used to assess the participants, clarify if a fasted condition was assured.

In “Data Collection” was included:

“For all methods used to assess the participants, was asked fasting for 12 hours previously”.

4. In the BIA description more details are required about the protocol (position, electrodes, etc).

5. Also, provide information about the raw BIA parameters that were assessed (resistance, reactance, PhA) and which frequency was used.

About this topics (4 and 5) it was included BIA description more details:

Body composition was assessed by using the bioelectrical impedance multiple-frequency (BIS) analysis (Body Composition Monitor – Fresenius Medical Care®), with different frequencies (5 to 1,000 kHz), and were considered the values obtained in 50 kHz. Patients were in supine decubitus, at rest 15 minutes before the measurement allowing a balance of body fluids with arms and legs abducted within a 30–45° angle from the trunk and electrodes to be affixed to the right hand and foot. BIS was calibrated regularly as an electronic verification module (usually provided by the manufacturer of this device). Other recommendations: refrain from any intense physical activity four hours prior to measurement, ensure that no metals are in the clothing, clean the skin with alcohol before placement of the electrodes.

The electrodes will be positioned as follows: an opposite pair next to breast cancer surgery, being a distal electrode at the base of the middle finger and the proximal between the medial and lateral malleoli, away from 5 cm between them; The other pair will be positioned in the contralateral hand the breast cancer surgery, with the distal electrode at the base of the middle finger and the proximal electrode coinciding with the style of style, also with a distance of 5 cm (Lukaski, 1987).

The BIS analysis provided data regarding resistence (R), reactance (Xc), body cell mass (BCM), fat mass (FM), fat-free mass (FFM), phase angle (PhA), total body water (TBW), extracellular water (ECW) and intracellular water (IW).

Reference: Lukaski HC. Methods for the assessment of human body composition: Traditional and new. Am J Clin Nutr. 1987;46(4):537–56.

6. Please include the precision (coefficient of variation-CV, for instances) for body composition variables and specifically for the PhA. The authors mentioned the precision for the biochemical variables but did not state which measurement was used (was it the CV?) and was it the same across all variables?

All biochemical data were previously laboratory tested, with CV below 5%.

The accuracy of BIS has been demonstrated elsewhere.

Reference: Moissl UM, Wabel P, Chamney PW et al. Body fluid volume determination via body composition spectroscopy in health and disease. Physiological Measurement 2006; 27: 921-9335.

7. Please include a reference that supports the expected effect size of 0.35.

According to Cohen’s guidelines, f2 ≥ 0.02, f2 ≥ 0.15, and f2 ≥ 0.35 represent small, medium, and large effect sizes, respectively.

The sample size calculation was performed using G*Power software version 3.1.9.4, taking into consideration the effect of independent variables on PhA (Li X et al., 2018).

This study was a post hoc analysis of data from an ongoing study which aim to explore possible changes in body composition, metabolic and oxidative stress parameters (Silva et al;. 2022).

References:

Selya AS, Rose JS, Dierker LC, Hedeker D, Mermelstein RJ. A Practical Guide to Calculating Cohen's f(2), a Measure of Local Effect Size, from PROC MIXED. Front Psychol. 2012 Apr 17;3:111. doi: 10.3389/fpsyg.2012.00111. PMID: 22529829; PMCID: PMC3328081.

Li X, Liu Z-l, Wu Y-t, Wu H, Dai W, Arshad B, Xu Z, Li H, Wu K-n, Kong L-q, et al. Status of lipid and lipoprotein in female breast cancer patients at initial diagnosis and during chemotherapy. Lipids Health Dis. 2018;17(1):1–6. doi:10.1186/s12944-018-0745-1.

Silva BR, Rufato S, Mialich MS, Cruz LP, Gozzo T, Jordao AA. (2022): An Evaluation of Metabolic, Dietetic, and Nutritional Status Reveals Impaired Nutritional Outcomes in Breast Cancer Patients. Nutr Cancer. 2022;74(10):3611-3622. https://doi: 10.1080/01635581.2022.2093388.

8. If the authors are interested in exploring how PhA could be a simple marker of health-related parameters (in this case oxidative stress and inflammation), the multiple regression analysis should consider PhA as the independent variable and not the dependent variable. The authors want to test the potential usefulness of PhA in tracking health-related parameters after adjusting for confounding factors (age, weight and height), because it is a simple marker, and not the other way around. Please make the required changes in the results and discussion.

The authors consider PhA as a dependent variable. Its value depends on changes in independent variables such as body composition, nutritional, biochemical and oxidative stress parameters.

Minor comment: please correct the word "statically" throughout the manuscript.

The word "statically" was replaced by “Statistically” throughout the manuscript.

**REVIEWER 2**

Reviewer #2: The manuscript is generally well written although in places the English is not entirely clear. Some examples are noted below. A thorough reading and sub-editing would be beneficial. Points to be considered.

Intro, para 1, line 5 "pathogenesis"

This word has been corrected.

Intro, para 2, line 2 "that triggers inflammatory"

This word has been corrected.

Intro, [para 3, line 3 "the bad outcomes related to the aftertreatment, especially regarding metabolic alterations ". This is not clearly worded - what is actually meant by "after-treatment"? An example of a metabolic alteration would be useful.

This part was supplemented by: “the bad outcomes related to the after-treatment, especially regarding metabolic alterations, **eg: risk for cardiovascular disease, metabolic syndrome** [19–23]”.

Methods, population, para 3, last line Please provide some details about the control group recruitment. Were the age, sex-matched etc.?

This topic was explained in item 2 of Reviewer 1.

Data collection, para 1. Please provide some information in relation to time of diagnosis, surgical treatment etc., presence of lymphedema (known to effect impedance measurements)....

No patient in the breast cancer group had lymphedema.

Anthropometric measurements. To what resolution were these measurements made, in replicate...? More detail required.

The anthropometric measurements were recorded as the average of three consecutive measures.

BIA. BIA is an indirect assessment method that is known to be population specific and requires careful standardization of the measurement procedures (see doi: 10.1080/03091902.2017.1333165. this is in children but the same principles apply in all age groups). Please provide details. The device used is a BIS device, hence a PhA is available at all measurement frequencies. Make it clear that this study is using PhA at 50 kHz (I assume!).

Thank you so much for this great paper!

This topic was explained in detail in itens 4 and 5 of Reviewer 1.

Statistical analysis, last para. "t-test" not "T-test". It is not entirely clear but it seems that the test group were assessed twice (To and T1) but the control group once only. In this case, the test group differences should be assessed using a paired-tests and the CG versus test group using a group analysis. A weakness of this study design is that the CG group were not followed for a similar time period as the test group. Thud change in the test group may simply be a reflection of normal fluctuation but unknown in the absence of comparable control data. This needs to be addressed and discussed as study limitation.

The authors included this description in the manuscript as a study limitation:

Study limitation

A limitation of the design of this study is that the GC group was not followed for a similar time (only T0 period) as the test group, with the absence of comparable control data. Although no significant change is expected in the data of the control group, as this group did not receive any type of intervention, the absence of repeated measures in the control group does not allow for the assessment of time-related changes in parameters in the test group, regardless of treatment or disease status.

Multiple regression analysis was used but what type, was it simple OLS or a derivative e.g. ridge regression or even a Bayesian approach. More details are required. For example which of the many potential variables were included and why? How was multi-collinearity assessed?

Statistical analysis, penultimate sentence. "To assess the ability of regressions models making predictions, it was used the verification by the least square methods". What exactly does this mean? Assessment of predictive power requires some form of cross-validation, e.g., split-grroup, LOOCV, K-means etc. This appears not to have been undertaken. Table 3 indicates that a particular set of variables may have some value in terms of explaining variability but this is not the same of developing a predictive model or algorithm. Please be more explicit in what you are wishing to achieve.

The method used is a simple OLS and the variables included were the body composition measures that clearly affect the PhA values and the other variables were included because the study aimed to examine the relationship between PhA and inflammatory and oxidative stress biomarkers.

So far, there is a lack of studies in the literature involving cancer patients and their relationship with PhA and oxidative stress, only 3 studies have explored this topic. This is an exploratory study and the first study involving cancer patients and their relationship with PA and oxidative stress, PhA is used as a dependent variable, in which it evaluated whether PhA was related to oxidative stress markers, that is, what were the determinants of PhA in our sample and whether oxidative stress markers were included in these determinants.

Results, Table 1. Please present all data to a consistent number of significant places, i.e., one. Also the the column headings, e.g., "T0xT1" requires more explanation. Are the values "t " values?, "P" values? Be consistent with units, e.g., both kg and KG are used. Use SI units. Abbreviations are not consistent, e.g., ECW and EX.

All these suggested changes have been included in Table 1.

The BIA data do not seem to be consistent. For example, mean FFM at T0 = 34 kg for CG = 35.6 kg, The corresponding TBW values are 31. 9 and 33.4 L. BIA is a 2C model and is typically calibrated against deuterium dilution to provide TBW and then FFM calculate using the hydration fraction of FFM (0.732). THus the calculated FFM from TBW are 43.6 and 45.6 for CG. These are not the FFM values provided? Why the discrepancy? THis casts a cloud over the quality of the data. This must be explained.

This BIA device was calibrated every day before data collection. These data were provided by the equipament itself and were exported directly from it, with no possibility of typing errors.

BIA is a doubly indirect method of assessing body composition, so the results are estimated from equations and not measured like other direct (cadaver dissection) or indirect (DXA, CT, MRI) methods. According to the manufacturer's manual this equipment is calibrated based on the double-labeled water method (deuterium dilution for estimate TBW).

Some assumptions have to be considered to use BIA for body composition:

1. The human body is empirically composed of cylinders, i.e., four limbs and trunk, with a uniform electric conductivity;

2. FFM contains virtually all the water and conducting electrolytes in the body;

3. FFM hydration is constant;

4. Conductive length is conventionally considered stature.

The 2-compartment model is based on an FFM density of 1.10 g/cm3, which was derived from an assumed FFM water fraction of 0.732 and various biological factors can influence FFM hydration. Apart from normal biological variation in hydration, there are strong indications that the relative amount of water in the FFM may be higher (FFM > 73%) in obese subjects (Waki et al., 1991).

This hydration constant of the fat-free mass (73.2%) has been criticized in the scientific literature long time ago (Wang et al., 1999) and this is one of the main limitations of the use of BIA in clinical practice. In overweight/obesity individuals, which is the case of the sample of this study, organ proportions may change with increasing body mass and, additionally, FFM hydration may be increased (Wang et al. 1999), so this hydration constant FFM tends to fail.

Waki et al., 1991 demonstrad that extracelular fluid is relatively increased in obese women suggest that the indirect estimation of body composition, based on assumptions that water distribuition is constant and independet of fatness, may produce greater error in some measurements of obese individuals. Finally, the authors concluded that TBW, extracelular fluid, intracelular fluid, and ratio of extracelllar to intracelular fluid were significantly increased in healthy obese women compared with nonobese women.

Recently studies agree that in obesity, a higher hydration of FFM is due to an increased water fraction of FFM in adipose tissue. For obesity, these effects are not adequately accounted for in the original BIA equations. The validity of BIA for body composition analysis in obesity is challenged by the assumption of a constant hydration of FFM that is violated by the higher hydration of FFM in adipose tissue and the higher ratio of extracellular (ECW) to intracellular water (ICW) in the adipose tissue part of connective tissue (Jensen et al., 2019).

Although, the estimation of body composition by BIA has been criticized by several authors its limited accuracy (i.e. body fat, fat-free mass), especially in a population with obesity however, PhA has been shown as a useful tool in several clinical conditions as it is not depends on prediction equations.

The focus of the present study is not on the raw quantification of FM and FFM, nor on diagnosing obesity and these body composition data are not being used to assess sarcoepnia, for example. The main objective of this study is to use the PhA, which is a raw measure of BIA, and not an estimated measure and does not depend on equations, and thus propose a new use for this data, possibly as a general marker of health and its association with oxidants and antioxidants markers.

Reference:

Sergi G, De Rui M, Stubbs B, Veronese N, Manzato E. Measurement of lean body mass using bioelectrical impedance analysis: a consideration of the pros and cons. Aging Clin Exp Res. 2017 Aug;29(4):591-597. doi: 10.1007/s40520-016-0622-6. Epub 2016 Aug 27. PMID: 27568020.

Wang Z, Deurenberg P, Wang W, Pietrobelli A, Baumgartner RN, Heymsfield SB. Hydration of fat-free body mass: review and critique of a classic body-composition constant. Am J Clin Nutr. 1999 May;69(5):833-41. doi: 10.1093/ajcn/69.5.833. PMID: 10232621.

Wang Z, Deurenberg P, Wang W, Pietrobelli A, Baumgartner RN, Heymsfield SB. Hydration of fat-free body mass: new physiological modeling approach. Am J Physiol. 1999 Jun;276(6):E995-E1003. doi: 10.1152/ajpendo.1999.276.6.E995. PMID: 10362610.

Waki M, Kral JG, Mazariegos M, Wang J, Pierson RN Jr, Heymsfield SB. Relative expansion of extracellular fluid in obese vs. nonobese women. Am J Physiol. 1991 Aug;261(2 Pt 1):E199-203. doi: 10.1152/ajpendo.1991.261.2.E199. PMID: 1872382.

Jensen B, Braun W, Geisler C, Both M, Klückmann K, Müller MJ, Bosy-Westphal A. Limitations of Fat-Free Mass for the Assessment of Muscle Mass in Obesity. Obes Facts. 2019;12(3):307-315. doi: 10.1159/000499607. Epub 2019 May 27. PMID: 31132777; PMCID: PMC6696776.

Discussion. The authors discuss the relationship between PhA and various variables in their study and those of others. They are, however, not duly critical that these are simply empirically-observed relationships that may have tenuous biological or physiological links. Although, not specifically stated I assume that the data are whole body (wrist to ankle) impedance data. Thus the PhA value represents some form of average value arising from all cells and structures along the conductive path. This will be all variety of tissue types including, muscles, organs etc. Consequently, it is difficult to see a real (causal?) connection between PhA and, for example, circulating albumin. This point needs to be made. Study limitations are not discussed, particularly, the absence of repeat measurements in the control group to allow for time-related changes in parameters in the test group irrespective of treatment or disease status.

Thanks for your comment. You are sure, the data are whole body (wrist to ankle) impedance data and you are correct the PhA value represents the average value arising from all cells and structures along the conductive path (diferente tissues, eg: muscles, organs, etc).

This discussion on the connection of PhA and circulating albumin was included:

“The PhA concept is based on changes in resistance and reactance as alternating current passes through evaluated tissues. Therefore, the measured PhA depends on several biological factors such as the quantity of cells with their respective cell membranes, cell membrane integrity, and related permeability and the amounts of extracellular and intracellular fluids (Gonzalez et al., 2016).

Therefore, the alterations in the PhA associated with malnutrition, specially early phases of malnutrition that FFM loss has not still occurred, extracellular fluid expansion can lead to an increase in the ECW:ICW ratio leading to a decrease in the PhA (Gonzalez et al., 2016).

Cornejo-Pareja et al (2021) demonstrate that a cut-off PhA value less tha 3.94 (ROC curve of survival) is more sensitive prognostic fator in predicting mortality in COVID-19 patients than standard biochemical measurements of inflammation such as ferritin, prealbumin, albumin, C-reactive protein (CRP). The authors highlight that this results, on the general COVID-19 population and not limited only to critical patients, may have greater applicability in clinical practice. Additionally, the survival analysis revealed 2.48 times higher hazards ratio of mortality for a decrease in 1° in PhA value.

Reference:

Cornejo-Pareja I, Vegas-Aguilar IM, García-Almeida JM, Bellido-Guerrero D, Talluri A, Lukaski H, Tinahones FJ. Phase angle and standardized phase angle from bioelectrical impedance measurements as a prognostic factor for mortality at 90 days in patients with COVID-19: A longitudinal cohort study. Clin Nutr. 2021 Feb 17:S0261-5614(21)00091-1.

The session “Strengths and limitations” has been included in the manuscript as described above.
