# Supplementary material for: Phase angle is related to oxidative stress and antioxidant biomarkers in breast cancer patients undergoing chemotherapy
Source: PLoS One. 2023 Jun 8;18(6):e0283235. doi: 10.1371/journal.pone.0283235 (PMC10249836; doi:10.1371/journal.pone.0283235)
Supplement: Attachment — Submitted filename: Response to Reviewers (January 26).docx [file pone.0283235.s003.docx]

Thank you very much for this more opportunity to review our manuscript “**Phase angle is related to oxidative stress and antioxidant biomarkers in breast cancer patients undergoing chemotherapy”**.

We completely agree with the review’s concerns. And due to the discrepancy between the TBW and FFM, we tried to contact the BIA’s manufacturer to check in every possible way which equation was built into the BIA’s system to estimate FM and FFM.

Unfortunately, this information is not disclosed in the manual. For this reason, we calculated FM and FFM from BIA’s raw data and the Kotler equation (1996), as described in detail below.

Please find below a point-by-point response to reviewer’s comments.

**JOURNAL REQUIREMENTS:**

Reviewer #1: The authors addressed the raised comments. The paper was improved and is now able to provide additional insights to the related research field.

Thank you again for this opportunity. We believe that all reviewer suggestions have substantially contributed to a better presentation of our findings.

Reviewer #2: The authors have addressed most of the issues raised but I remain concerned about the BIA data in Table 1, i.e., the discrepancy between the stated TBW and FFM values. The authors have provided a brief but comprehensive review of the BIA methods and made reference to differences in hydration fraction in different populations. But this does not address the key issue. Going back to my example from the Table, for a TBW of 31.9 L and an FFM of 34 kg suggests an HF of 31.9/34 or 93.8%, physiologically unrealistic. Hydration values such as this are simply not seen.

Something is wrong here. I may be misinterpreting the data but I do not think so. To state that the papers focus is not on FM and FFM is missing the point. If these are not correct then how can the reader be assured that the phase data are correct since FFM and FM have been calculated from the measured BIA data. This MUST be explained for, at least, this reader to have confidence in data validity.

Thank you for these important appointments on the BIA data (Table 1).

BIA is a doubly indirect method which uses predictive equations to estimate body composition derived from comparisons with reference methods. Most studies using BIA to predict body composition in the last years developed many equations.

Talking specifically of the device used in this study, we used the FM and FFM obtained directly from the BIA. We checked the manual and consulted the Fresenius website to confirm which equation the software uses; however, we could not verify that.

In this sense, our research group entirely agrees with the reviewer's comments. Instead of using the FM and FFM obtained from the device, we correct it by using the BIA's raw data and the predictive equation developed by Kotler et al. (1996).

Kotler et al. (1996) proposed a new BIA formula validated against DXA for white and non-white subjects, including a group of HIV-positive patients, which used logarithmic transformation of height, reactance, and impedance and found them to be more accurate predictors than equations using height^2^/resistance.

Going back to the relevant reviewer's comments, BIA is a 2C model and is typically calibrated against deuterium dilution to provide TBW and then FFM calculate using the hydration fraction of FFM (0.732). The corresponding TBW values are 31.9 L, 33,2 L and 33.4 L, respectively in T0, T1 and CG.

Therefore, FFM and FM have been calculated from predictive equation (Kotler et al., 1996) and the final values obtained are perfectly compatible with the values expected by the BIA and correspond to the acceptable hydration fraction (HF) according to the principles of this method.

|  | Kotler et al. (1996) | | | BIS | |
| --- | --- | --- | --- | --- | --- |
|  | **FFM _Equation_ (kg)** | **FM _Equation_ (kg)** | **HF** | **FFM _BIS_ (kg)** | **FM _BIS_ (kg)** |
| T0 | 45,1 (SD 5,1) | 27,6 (SD 10,3) | 0,705 (SD 0,05) | 34 (SD 7,1) | 28,82 (SD 9,09) |
| T1 | 47,5 (SD 6,8) | 27,3 (SD 10,5) | 0,697 (SD 0,08) | 32,5 (SD 5,6) | 28,78 (SD 8,94) |
| CG | 46,8 (SD 5,6) | 29,6 (15,6) | 0,711 (SD 0,07) | 35,62 (SD 6,3) | 30,23 (SD 13,82) |

All these new FFM and FM values were incorporated in the manuscript, as well as their respective updated p values - **Manuscript (January 26)** and **Revised Manuscript with tracked changes (January 26).**

We hope this effort will clarify these important points. We are really honored by this review and the opportunity to study and reflect further on our study’s data.

[1] Kotler DP, Burastero S, Wang J, Pierson RN. Prediction of body cell mass, fatfree mass, and total body water with bioelectrical impedance analysis: effects of race, sex, and disease. Am J Clin Nutr 1996;64(suppl). 489S-97S.
