# Supplementary material for: Phase angle is related to oxidative stress and antioxidant biomarkers in breast cancer patients undergoing chemotherapy
Source: PLoS One. 2023 Jun 8;18(6):e0283235. doi: 10.1371/journal.pone.0283235 (PMC10249836; doi:10.1371/journal.pone.0283235)
Supplement: Attachment — Submitted filename: Response to reviewer (Feb 17).docx [file pone.0283235.s004.docx]

Thank you very much for the opportunity to review our manuscript “Phase angle is related to oxidative stress and antioxidant biomarkers in breast cancer patients undergoing chemotherapy.”.

Please find below the response to reviewer’s comment. We hope to have satisfactorily addressed concerns and suggestions.

Reviewer #2:

Thank you for now calculating FFM according to the Kotler equation. I do not wish to delay the paper further but the issue of the incorrect BIS TBW and FFM remains. The authors are correct about BIA being a 2C model etc., thus it is disturbing that BIS_FM + BIS_FFM do not equal weight (Table 1: 34 + 28.8 = 62.8 yet weight is 71.7 kg).

I wonder if this is a nomenclature issue and that BIS-FFM is actually BIS_Lean, i.e. FFM - BMC. This could be the explanation. I suggest that the authors consult the device manual to check this.

We would like to thank you, the reviewer, for the opportunity to review our data further. Based on the reviewer’s comment, we have reviewed the Fresenius Medical Care Body Composition Monitor manual and website; indeed, there was a nomenclature issue. Fresenius Body Composition Monitor describes that the device provides information regarding Lean Tissue Mass and not Fat-free mass [1]. The device estimates the Lean Tissue Mass from Extracellular Water and Total Body Water information [1].

Thank you for bringing this to our attention. Throughout the manuscript, we have corrected this by replacing Fat-free Mass (FFM) obtained by the BIS device with the appropriate nomenclature: Lean Tissue Mass (LTM).

The information regarding Lean Tissue Mass can be found and confirmed at:

<https://www.freseniusmedicalcare.com/en/body-composition-monitor>

<https://www.freseniusmedicalcare.com/fileadmin/data/masterContent/pdf/Healthcare_Professionals/Fluid_Management/BCM_Technical_Data.pdf>

References

[1]	Fresenius Medical Care. BCM - Body Composition Monitor. https://www.freseniusmedicalcare.com/en/body-composition-monitor (accessed February 16, 2023).
